# Measurement of absolute frequency of continuous-wave terahertz radiation in real time using a free-running, dual-wavelength mode-locked, erbium-doped fibre laser

**DOI:** 10.1038/srep42082

**Published:** 2017-02-10

**Authors:** Guoqing Hu, Tatsuya Mizuguchi, Xin Zhao, Takeo Minamikawa, Takahiko Mizuno, Yuli Yang, Cui Li, Ming Bai, Zheng Zheng, Takeshi Yasui

**Affiliations:** 1 School of Electronic and Information Engineering, Beihang University, Beijing, 100191, China; 2 Graduate School of Science and Technology, Tokushima University, 2-1 Minami-Josanjima, Tokushima 770-8506, Japan; 3 JST, ERATO, MINOSHIMA Intelligent Optical Synthesizer Project, 2-1 Minami-Josanjima, Tokushima 770-8506, Japan; 4 Collaborative Innovation Centre of Geospatial Technology, 129 Luoyu Road, Wuhan 430079, China

## Abstract

A single, free-running, dual-wavelength mode-locked, erbium-doped fibre laser was exploited to measure the absolute frequency of continuous-wave terahertz (CW-THz) radiation in real time using dual THz combs of photo-carriers (dual PC-THz combs). Two independent mode-locked laser beams with different wavelengths and different repetition frequencies were generated from this laser and were used to generate dual PC-THz combs having different frequency spacings in photoconductive antennae. Based on the dual PC-THz combs, the absolute frequency of CW-THz radiation was determined with a relative precision of 1.2 × 10^−9^ and a relative accuracy of 1.4 × 10^−9^ at a sampling rate of 100 Hz. Real-time determination of the absolute frequency of CW-THz radiation varying over a few tens of GHz was also demonstrated. Use of a single dual-wavelength mode-locked fibre laser, in place of dual mode-locked lasers, greatly reduced the size, complexity, and cost of the measurement system while maintaining the real-time capability and high measurement precision.

Terahertz (THz) radiation covers an extremely wide electromagnetic band that potentially could be leveraged for high-speed communications, and investigation of THz radiation has attracted increasing interest^1^,^2^. With the development of various continuous-wave THz (CW-THz) radiation sources, such as THz quantum cascade lasers^3^ and uni-traveling-carrier photodiodes^4^, THz wireless communication is highly promising, even though frequency allocation in the THz band (0.275–3 THz) has not yet been established. For the purpose of evaluating sources and considering suitable frequency allocation, it is essential to precisely determine the absolute frequency of CW-THz radiation. Although the electrical heterodyne method^5^ and the optical interferometric method have been used for measuring the absolute frequency of CW-THz radiation, both of these methods need cryogenic cooling to reduce thermal noise, hindering the wide adoption of these methods in various practical applications. Therefore, there is a strong demand for absolute frequency measurement in the THz region without the need for cryogenic cooling.

One promising method of achieving this is the scheme based on photoconductive mixing of CW-THz radiation with a THz frequency comb of a photo-carrier (PC-THz comb) in a photoconductive antenna (PCA)^6^,^7^,^8^,^9^,^10^,^11^. In this scheme, the absolute frequency *f*_*THz*_ of CW-THz radiation can be determined from a PC-THz comb mode *m* nearest in frequency to *f*_*THz*_, the frequency interval *f*_*rep*_ of the PC-THz comb, and the beat frequency *f*_*beat*_ between the CW-THz radiation and the *m*-th comb mode. While *f*_*rep*_ and *f*_*beat*_ can be directly measured in the radio-frequency (RF) region, *m* can be determined by two different *f*_*rep*_ values and their corresponding *f*_*beat*_ values. In early research^6^,^7^,^8^, the determination of *m* was based on time-sequential, two-step measurement of *f*_*rep*_ and *f*_*beat*_ with a single PC-THz comb induced by an *f*_*rep*_-adjustable mode-locked laser. Therefore, *f*_*THz*_ could not be determined in real time. Recently, dual PC-THz combs with different *f*_*rep*_ have been used to achieve the real-time determination of *f*_*THz*_ based on simultaneous measurement of two *f*_*rep*_ values and their corresponding *f*_*beat*_ values^10^. In that study, by using dual stabilized or free-running mode-locked lasers with different *f*_*rep*_ for the generation of dual PC-THz combs, *f*_*THz*_ was determined precisely at a measurement rate of 100 Hz. However, use of dual laser systems hinders the wider adoption of such techniques. More recently, the real-time determination of *f*_*THz*_ was achieved by using a single mode-locked laser with an actively modulated or smoothly drifting *f*_*rep*_^11^; however, the measurement rate remained at 10 Hz due to the time-sequential, fast-two-step measurement of *f*_*rep*_ and its corresponding *f*_*beat*_ with a single PC-THz comb. If dual PC-THz combs with different *f*_*rep*_ could be generated by a single free-running mode-locked laser, the real-time capability, precision, and practicability of THz frequency measurement would be enhanced.

Recently, the use of ‘multiplexed’ mode-locked erbium-doped fibre (Er:fibre) lasers as dual-comb lasers has been demonstrated by multiplexing in the dimensions of centre wavelength, propagation direction, polarization state, or mode-locking mechanism^12^,^13^,^14^,^15^,^16^,^17^. Among these schemes, use of a dual-wavelength (dual-λ) mode-locked Er:fibre laser is a promising way to generate a dual PC-THz comb because it emits two independent mode-locked pulsed light beams with different wavelengths, *λ*_*1*_ and *λ*_*2*_, from a single cavity, and their *f*_*rep*_ values are slightly detuned from each other due to dispersion in the fibre laser cavity^18^. The pulsed light beams with wavelengths *λ*_*1*_ and *λ*_*2*_ can be easily separated by optical filters, and the difference in *f*_*rep*_ between them can be adjusted by dispersion management in the fibre cavity. Also, the common-mode noise between the *λ*_*1*_ and *λ*_*2*_ pulsed beams is effectively cancelled by co-propagation of them in the same cavity^19^. Such characteristics in dual-*λ* mode-locked Er:fibre lasers have been successfully used in asynchronous optical sampling (ASOPS) pump-probe measurement^18^, optical ranging^20^, and optical spectroscopy^19^. However, there have been no attempts to apply the technique to THz measurement. In this paper, we used a dual-*λ* mode-locked Er:fibre laser for rapid, high-precision measurement of *f*_*THz*_ based on dual PC-THz combs.

## Principle of Operation

THz-comb-referenced frequency measurement is based on heterodyne photoconductive mixing between CW-THz radiation and a PC-THz comb^6^,^7^. Two essential conditions must be satisfied: (1) a PCA must work as a broadband heterodyne receiver with high sensitivity for THz radiation at room temperature, and (2) the generated PC-THz comb should cover the whole THz band. When CW-THz radiation (freq. = *f*_*THz*_) is photoconductively mixed with one mode of a single PC-THz comb (freq. interval = *f*_*rep*_, comb mode nearest in frequency to *f*_*THz*_ = *m*), *f*_*THz*_ is given by


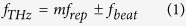


where *f*_*beat*_ is the beat frequency between CW-THz radiation and the *m*-th comb mode.

Next we consider the photoconductive mixing of CW-THz radiation with dual PC-THz combs having different frequency spacings (PC-THz comb 1, freq. interval = *f*_*rep1*_, comb mode nearest in frequency to *f*_*THz*_ = *m*; PC-THz comb 2, freq. interval = *f*_*rep2*_, comb mode nearest in frequency to *f*_*THz*_ = *m*). In this case, when *f*_*rep2*_* *>* f*_*rep1*_, *f*_*THz*_ is given by


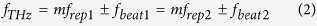


where *f*_*beat1*_ is the beat frequency between the CW-THz radiation and the *m*-th comb mode in PC-THz comb1, and *f*_*beat2*_ is the beat frequency between the CW-THz radiation and the *m*-th mode in PC-THz comb2. From Eq. (2), *m* can be calculated by


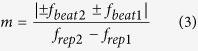


where the signs of *f*_*beat1*_ and *f*_*beat2*_ are determined by the relative positions of *f*_*THz*_, *mf*_*rep1*_, and *mf*_*rep2*_.

**Figure 1:**
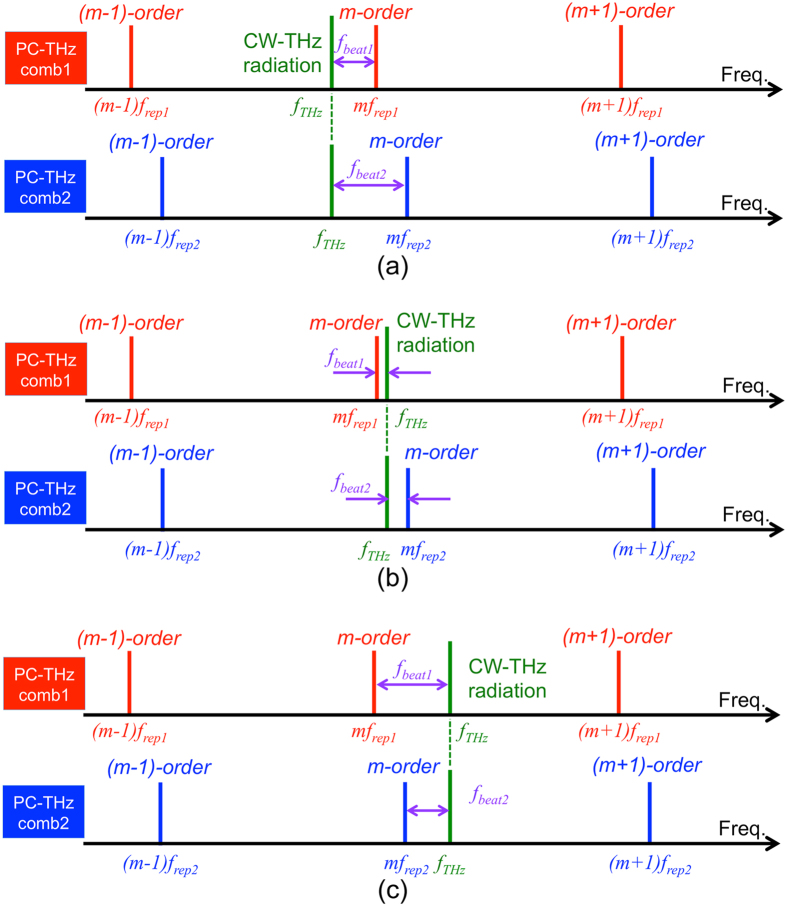
Relative positions of *f*_*THz*_, m*f*_*rep1*_, and *mf*_*rep2*_. (**a**) *f*_*THz*_ < *mf*_*rep1*_* < mf*_*rep2*_, (**b**) *mf*_*rep1*_* < f*_*THz*_* < mf*_*rep2*_, and (**c**) *mf*_*rep1*_* < mf*_*rep2*_* < f*_*THz*_.

Figure 1 shows the relative position of *f*_*THz*_ (see green line) to the nearest modes *mf*_*rep1*_ (see red lines) and *mf*_*rep2*_ (see blue lines) in the dual PC-THz combs, where (a) *f*_*THz*_ < *mf*_*rep1*_* < mf*_*rep2*_, (b) *mf*_*rep1*_* < f*_*THz*_* < mf*_*rep2*_, and (c) *mf*_*rep1*_* < mf*_*rep2*_* < f*_*THz*_. Since the frequency difference between *f*_*rep1*_ and *f*_*rep2*_ ( = *f*_*rep2*_ − *f*_*rep1*_ = *∆f*_*rep*_) is the denominator of Eq. (3), a highly stable frequency difference is essential for accurately determining *m*. The relative positions of *f*_*THz*_, *mf*_*rep1*_, and *mf*_*rep2*_ can be determined from the simultaneous measurements of *f*_*rep1*_, *f*_*rep2*_, *f*_*beat1*_, and *f*_*beat2*_ as follows:


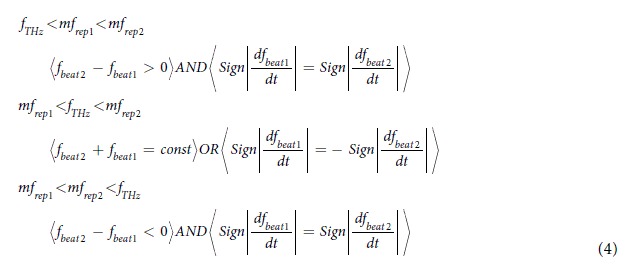


Therefore, *m* can be obtained by


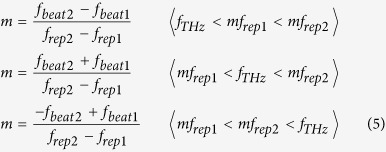


Finally, *f*_*THz*_ can be determined by


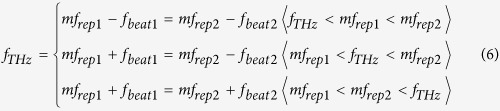


## Results

### Free-running, dual-*λ* mode-locked Er:fibre laser

**Figure 2:**
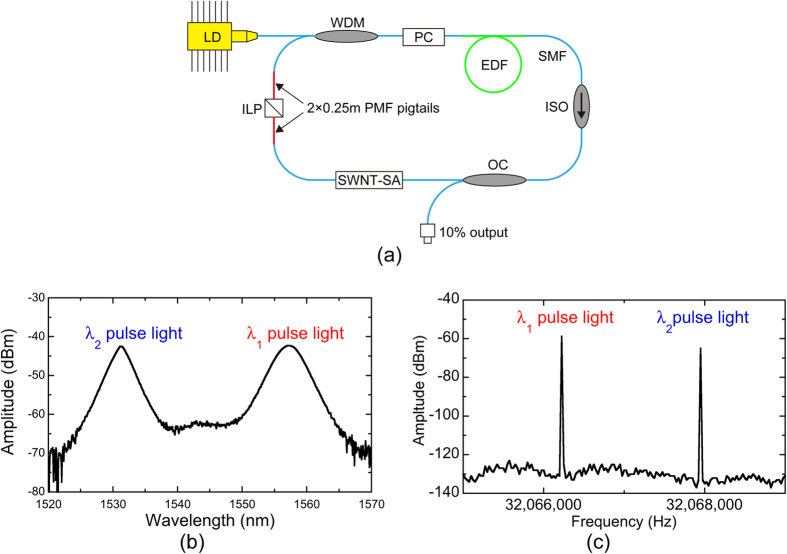
(**a**) Configuration of free-running dual-λ mode-locked Er-doped fibre laser oscillator. LD, 980-nm pump diode; WDM, 980/1550 nm wavelength-division multiplexer; SMF, single-mode fibre; EDF, erbium-doped fibre; ISO, polarization-independent optical isolator; SWNT-SA, single-wall carbon nanotube saturable absorber; PC, fibre-squeezer-based polarization controller; OC, 90/10 fibre output coupler; ILP, in-line polarizer; PMF, polarization maintaining fibre. (**b**) Optical spectrum and (**c**) RF spectrum of the output light from the laser oscillator.

Figure 2(a) shows the configuration of the free-running, dual-λ mode-locked Er:fibre laser oscillator. With birefringence-induced filtering and loss control effects^12^,^13^,^19^, in addition to the adjustment of the polarization state in the ring cavity, simultaneous mode-locking centred on the 1530-nm and 1560-nm regions can be realized. As shown in Fig. 2(b), the centre wavelengths of dual-λ pulses were 1531.4 nm and 1556.1 nm, with corresponding 3-dB bandwidths of 2.2 nm and 3.3 nm, respectively. Because of the anomalous intracavity dispersion, the dual-λ pulses had different repetition rates around 32.06 MHz (*f*_*rep1*_ = 32,066,206 Hz, *f*_*rep2*_ = 32,067,857 Hz) with a difference *∆f*_*rep*_ ( = *f*_*rep2*_ − *f*_*rep1*_) of ~1.63 kHz, as shown in Fig. 2(c).

**Figure 3:**
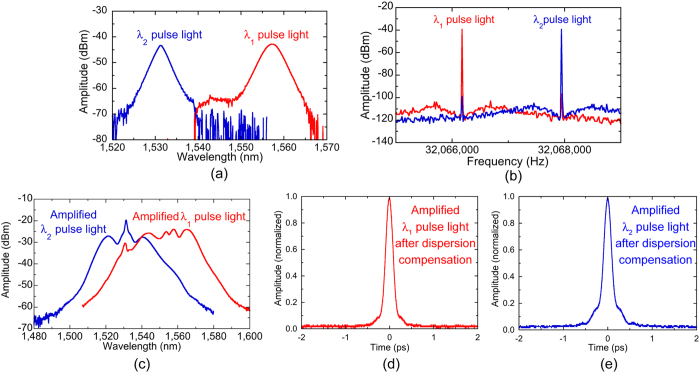
(**a**) Optical spectra and (**b**) RF spectra of the *λ*_*1*_ and *λ*_*2*_ pulsed light after passing through the CWDM bandpass filter. (**c**) Optical spectra of the amplified *λ*_*1*_ and *λ*_*2*_ pulsed light. (**d**) and (**e**) Auto-correlation waveforms of the amplified *λ*_*1*_ and *λ*_*2*_ pulsed light after dispersion compensation.

In order to meet the optical power and pulse duration requirements for PCAs, the *λ*_*1*_ and *λ*_*2*_ pulses from the laser oscillator were separated by a coarse-wavelength-division-multiplexing bandpass filter (CWDM-BPF) [not shown in Fig. 2(a)]. Figure 3(a and b) show optical spectra and RF spectra of the *λ*_*1*_ and *λ*_*2*_ pulses after passing through the CWDM bandpass filter. The dual-λ mode-locked fibre laser light was successfully separated into each component in the optical region and the RF region. Then, the components were amplified and spectrally broadened by erbium-doped fibre amplifiers (EDFAs) and the following SMF, respectively. As shown in Fig. 3(c), the optical spectrum of the amplified *λ*_*1*_ pulsed light covered the whole C band, whereas that of the amplified *λ*_*2*_ pulsed light was located at the shorter wavelength side. The mean power and the pulse duration were 27 mW and 130 fs for the amplified *λ*_*1*_ pulsed light [see Fig. 3(d)] and 20 mW and 130 fs for the amplified *λ*_*2*_ pulsed light [see Fig. 3(e)] when the SMF was used to compensate for the dispersion. These output characteristics were sufficient to generate a PC-THz comb in PCA.

**Figure 4:**
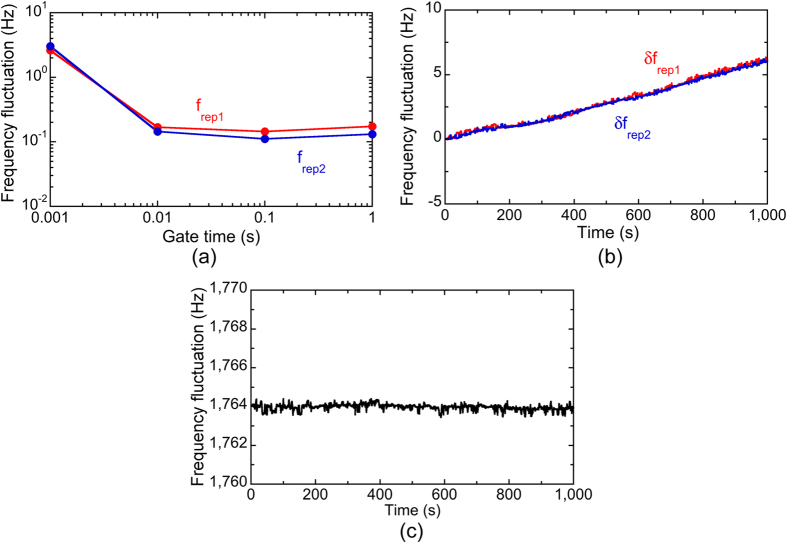
(**a**) Frequency fluctuations of *f*_*rep1*_ and *f*_*rep2*_ with respect to gate time. Temporal fluctuations of (**b**) *δf*_*rep1*_ and *δf*_*rep2*_ and (c) *∆f*_*rep*_.

Before performing real-time measurement of *f*_*THz*_ with dual PC-THz combs, we investigated the frequency characteristics of this free-running laser. We first measured the temporal fluctuations of *f*_*rep1*_ and *f*_*rep2*_ with a frequency counter (Agilent 53132 A). Figure 4(a) shows the fluctuations with respect to different gate times. Due to the free-running operation without active frequency control, the fluctuations of *f*_*rep1*_ and *f*_*rep2*_ did not decrease over a gate time of 0.1 s. However, these fluctuations were comparable to those of other commercialized, free-running single-wavelength lasers^10^,^11^; this is clear evidence that the two mode-locked operations at *λ*_*1*_ and *λ*_*2*_ do not compete with each other and are completely independent of each other. Figure 4(b) shows the temporal fluctuations of *f*_*rep1*_ and *f*_*rep2*_, where their frequency deviations from the initial values are indicated by *δf*_*rep1*_ and *δf*_*rep2*_. A slow drift was clearly confirmed for both, indicating changes in the environmental conditions in the fibre cavity. However, it should be emphasized that the temporal behaviours of *δf*_*rep1*_ and *δf*_*rep2*_ were the same. This is because the *λ*_*1*_ and *λ*_*2*_ pulses co-propagated in the same ring cavity and experienced similar disturbances. As a result of such common-mode behaviour of *δf*_*rep1*_ and *δf*_*rep2*_, *∆f*_*rep*_ was highly stable, as shown in Fig. 4(c). The mean and standard deviation of *∆f*_*rep*_ in Fig. 4(c) were 1764.97 Hz and 0.24 Hz, respectively. Such high stability of *∆f*_*rep*_ was useful for the correct determination of *m* and *f*_*THz*_ based on Eqs. (4 to 6). Therefore, even though *f*_*rep1*_ and *f*_*rep2*_ were not actively stabilized, this dual-λ mode-locked fibre laser can be used for measuring *f*_*THz*_ in real time and with high-precision using dual PC-THz combs.

### Real-time determination of *f*_*THz*_ with dual PC-THz combs

**Figure 5:**
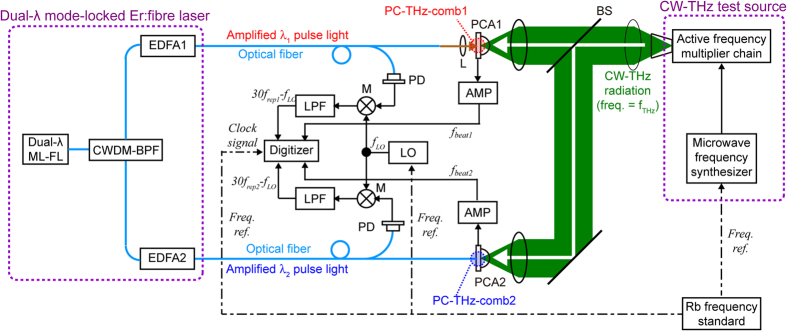
Experimental setup of THz-comb-referenced frequency measurement. Dual-λ ML-FL, free-running dual-λ mode-locked Er-doped fibre laser oscillator; CWDM-BPF, coarse-wavelength-division-multiplexing bandpass filter; EDFA1 and EDFA2, Er-doped fibre amplifiers; L, lens; PCA1, free-space-coupled, bowtie-shaped, low-temperature-grown (LT) InGaAs/InAlAs photoconductive antenna; PCA2, fibre-coupled, dipole-shaped LT-InGaAs/InAlAs PCA detector; PD, photodetectors; M, double-balanced mixer; LPF, low-pass filter; AMP, current preamplifiers; LO, local oscillator; BS, beam splitters.

Figure 5 shows a schematic diagram of the setup for measuring the frequency of CW-THz radiation, consisting of three main parts. The first part is the laser source, including a free-running, dual-λ mode-locked Er:fibre laser oscillator and two EDFAs. The second part is composed of the optical and THz systems for frequency measurement of CW-THz radiation, a CW-THz test source, a pair of low-temperature-grown (LT) InGaAs/InAlAs PCAs (PCA1 and PCA2), and their affiliated components. The third part is the data acquisition electronics.

The amplified *λ*_*1*_ pulsed light at *f*_*rep1*_ from one EDFA (EDFA1) was used for generating a PC-THz comb in PCA1 (PC-THz comb 1, freq. spacing = *f*_*rep1*_), whereas the amplified *λ*_*2*_ pulsed light at *f*_*rep2*_ from another EDFA (EDFA2) was used for generating a PC-THz comb in PCA2 (PC-THz comb 2, freq. spacing = *f*_*rep2*_). When the CW-THz radiation was incident on both PCA1 and PCA2, photoconductive mixing between the CW-THz radiation and the dual PC-THz combs and the following electronic processing resulted in the generation of beat signals with frequencies *f*_*beat1*_ and *f*_*beat2*_. On the other hand, RF signals related to *f*_*rep1*_ or *f*_*rep2*_ (freq. = 30*f*_*rep1*_ − *f*_*LO*_ and 30*f*_*rep2*_ − *f*_*LO*_) were obtained by the photodetectors (PD) and subsequent electric heterodyning with a local oscillator (LO, freq. = *f*_*LO*_). Temporal waveforms of *f*_*beat1*_, *f*_*beat2*_, 30*f*_*rep1*_ − *f*_*LO*_, and 30*f*_*rep2*_ − *f*_*LO*_ were simultaneously acquired by a digitizer (resolution = 14 bit, sampling rate = 20 MHz). From the temporal waveforms, we determined instantaneous values of *f*_*rep1*_, *f*_*rep2*_, *f*_*beat1*_, and *f*_*beat2*_ using the instantaneous-frequency-calculation algorithm^8^. Finally, we determined *f*_*THz*_ by substituting them into Eqs. (4 to 6). Since the CW-THz test source, the local oscillator, and the clock signals of the digitizer shared a common time-base signal from a 10 MHz rubidium (Rb) frequency standard (Stanford Research Systems FS725, accuracy = 5 × 10^–11^, stability = 2 × 10^−11^ at 1 s), one can evaluate the relative precision of frequency measurement without the influence of the absolute precision of the frequency standard.

**Figure 6:**
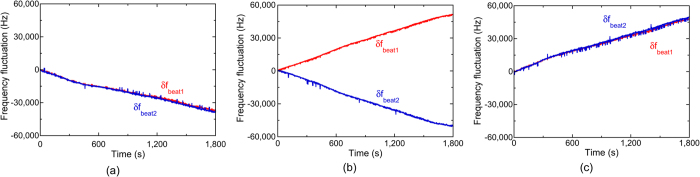
Temporal changes of *δf*_*beat1*_ and *δf*_*beat2*_ when (**a**) *f*_*THz*_ < *mf*_*rep1*_* < mf*_*rep2*_, (**b**) *mf*_*rep1*_* < f*_*THz*_* < mf*_*rep2*_, and (**c**) *mf*_*rep1*_* < mf*_*rep2*_* < f*_*THz*_.

To confirm the three situations in Fig. 1, we measured *f*_*beat1*_ and *f*_*beat2*_ when *f*_*THz*_ was set at (a) 100,013,820,000 Hz for *f*_*THz*_ < *mf*_*rep1*_* < mf*_*rep2*_, (b) 100,016,340,000 Hz for *mf*_*rep1*_* < f*_*THz*_* < mf*_*rep2*_, and (c) 100,020,240,000 Hz for *mf*_*rep1*_* < mf*_*rep2*_* < f*_*THz*_. Figure 6 shows the temporal change of *f*_*beat1*_ and *f*_*beat2*_, where their frequency deviations from the initial values are indicated by *δf*_*beat1*_ and *δf*_*beat2*_, when (a) *f*_*THz*_ < *mf*_*rep1*_* < mf*_*rep2*_, (b) *mf*_*rep1*_* < f*_*THz*_* < mf*_*rep2*_, and (c) *mf*_*rep1*_* < mf*_*rep2*_* < f*_*THz*_. In all graphs, *f*_*beat1*_ and *f*_*beat2*_ fluctuated monotonically due to the drift of *f*_*rep1*_ and *f*_*rep2*_ in the free-running operation. However, the directions of the temporal fluctuations were different from each other. In Fig. 6(a and c), *f*_*beat1*_ and *f*_*beat2*_ indicated similar behaviour to each other, namely, a monotonic decrease or increase. On the other hand, in Fig. 6(b), *f*_*beat1*_ and *f*_*beat2*_ changed in the opposite directions to each other, while their sum remained constant. These behaviours correctly reflect three situations in Fig. 1 and Eq. (4). Finally, we could correctly determine *m* to be all 3,119 in Fig. 6(a,b and c) based on Eqs (4 to 6).

**Figure 7:**
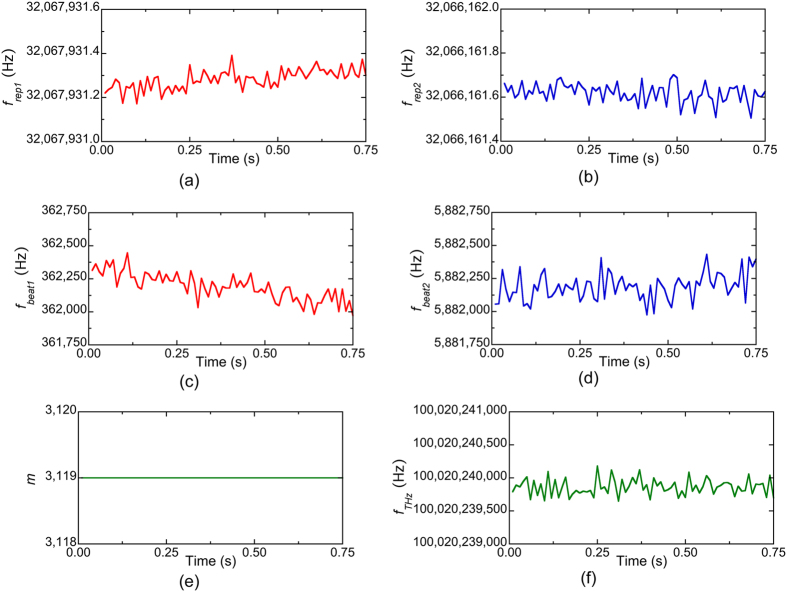
Temporal waveforms of (**a**) *f*_*rep1*_, (**b**) *f*_*rep2*_, (**c**) *f*_*beat1*_, (**d**) *f*_*beat2*_, (**e**) *m*, and (**f**) *f*_*THz*_ when *f*_*THz*_ was fixed at 100,020,240,000 Hz. The measurement rate was 100 Hz.

Next, we measured *f*_*rep1*_, *f*_*rep2*_, *f*_*beat1*_, and *f*_*beat2*_ when *f*_*THz*_ was fixed at 100,020,240,000 Hz. After acquiring the temporal waveforms for *f*_*rep1*_, *f*_*rep2*_, *f*_*beat1*_, and *f*_*beat2*_ at a sampling rate of 20 MHz, we calculated their mean values every 10 ms, which corresponds to a measurement rate of 100 Hz. Figure 7(a,b,c and d) show the temporal changes of the mean values for them. All values temporally fluctuated due to the free-running behaviour of the laser rather than the fluctuation of *f*_*THz*_. By substituting *f*_*rep1*_, *f*_*rep2*_, *f*_*beat1*_, and *f*_*beat2*_ in Eqs (4 to 5), the value of *m* was determined to be 3,119, as shown in Fig. 7(e). Finally, from Eq. (6), we determined the mean and standard deviation of *f*_*THz*_ to be 100,020,239,860 Hz and 125 Hz in repetitive measurements of *f*_*THz*_ at a measurement rate of 100 Hz, as shown in Fig. 7(f). Therefore, the relative accuracy and precision of the absolute frequency measurement were 1.4 × 10^−9^ and 1.2 × 10^−9^, respectively.

**Figure 8:**
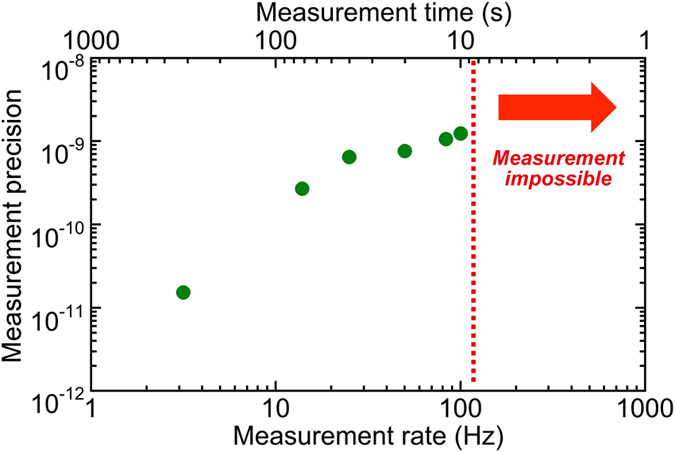
Measurement precision with respect to the measurement rate and the corresponding measurement time.

Figure 8 shows the measurement precision with respect to the measurement rate and the corresponding measurement time. The measurement precision and the measurement rate showed a trade-off relation within a range of measurement rates from 1 to 100 Hz. However, the correct determination of *f*_*THz*_ was impossible at measurement rates higher than 100 Hz, because the measurement error of the numerator | ± *f*_*beat2*_ ± *f*_*beat1*_| over the denominator *f*_*rep2*_ − *f*_*rep1*_ in Eq. (5) makes it impossible to determine *m* correctly.

**Figure 9:**
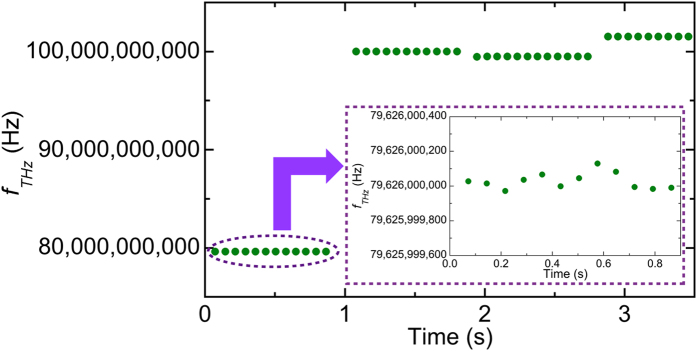
Real-time monitoring of *f*_*THz*_ when *f*_*THz*_ was suddenly or slightly changed. Inset shows the magnified fluctuation of *f*_*THz*_ from 0 s to 0.864 s.

Finally, we performed real-time monitoring of *f*_*THz*_ when *f*_*THz*_ was changed suddenly or slightly. Figure 9 shows the measured *f*_*THz*_ when the nominal frequency of the CW-THz test source was first set at 79,626,000,000 Hz, increased by 20,395,080,000 Hz, decreased by 513,120,000 Hz, and then increased by 2,020,260,000 Hz. The measured *f*_*THz*_ at each frequency setting was determined to be 79,626,000,029 ± 47 Hz, 100,021,079,989 ± 32 Hz, 99,507,959,988 ± 26 Hz, and 101,528,219,978 ± 36 Hz, respectively. Even though *f*_*THz*_ changed across many modes in the dual PC-THz combs, *f*_*THz*_ was determined correctly.

## Discussion

One may wonder why such high precision was achieved in the real-time measurement of *f*_*THz*_ by using the dual-PC-THz combs without the stabilization of *f*_*rep1*_ and *f*_*rep2*_. The reason is that each PC-THz comb always functions as a frequency ruler with equal intervals and a linear scale regardless of whether or not *f*_*rep1*_ and *f*_*rep2*_ are stabilized. Such characteristics are inherent in frequency combs. Only if the temporal waveforms for *f*_*rep1*_, *f*_*rep2*_, *f*_*beat1*_, and *f*_*beat2*_, are acquired synchronously, *f*_*THz*_ can be determined without the influence of unstabilized *f*_*rep1*_ and *f*_*rep2*_, as demonstrated in Figs 7(f) and 9.

The precision of 1.2 × 10^−9^ was achieved at a measurement rate of 100 Hz in the present setup; however, it was 100-times worse than that of the previous experiment with two independent free-running mode-locked lasers^10^. In the instantaneous-frequency-calculation algorithm^8^, the precision is largely influenced by the signal-to-noise ratio (SNR) of the beat signals with *f*_*beat1*_ and *f*_*beat2*_^10^. The beat signals measured by LT-InGaAs/InAlAs PCAs in the present setup showed the much lower SNR than the signals measured by LT-GaAs PCAs in the previous setup due to high dark-current noise in the LT-InGaAs/InAlAs PCAs (not shown). Therefore, the difference in precision between them arises from the low SNR in beat signals rather than use of the free-running dual-*λ* mode-locked Er:fibre lasers. In other words, there is still some room to enhance the precision by improving the PCAs.

**Figure 10:**
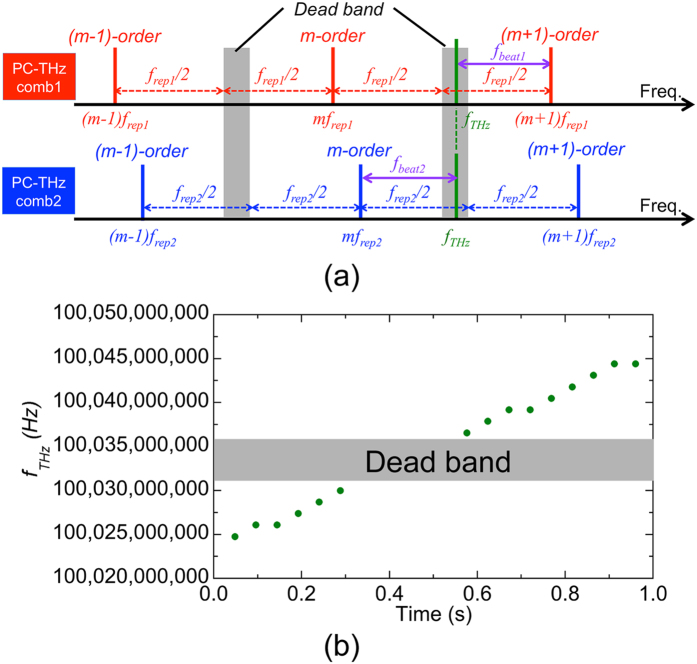
(**a**) Dead band of *f*_*THz*_ determination. (**b**) Measurement errors in the real-time monitoring of *f*_*THz*_ when *f*_*THz*_ is linearly tuned 100,024,770,463 Hz to 100,044,423,685 Hz.

*∆f*_*rep*_ (= 1.63 kHz) in the dual-λ mode-locked fibre laser used here was relatively high compared with that (typically, less than several tens Hz) in dual mode-locked lasers used in the previous research^10^. In this case, we cannot neglect the dead band in the determination of *m*. In Fig. 1 and Eqs. (1 to 6), it is assumed that the beat signals at the lowest frequency (freq. = *f*_*beat1*_ and *f*_*beat2*_) are generated by the same mode number *m* of dual PC-THz combs (freq. = *mf*_*rep1*_ and *mf*_*rep2*_). The dead band is generated when *f*_*beat1*_ and *f*_*beat2*_ are generated by different mode numbers of the dual PC-THz combs. Figure 10(a) shows the optical spectrum when *f*_*THz*_ exists within the dead band, namely


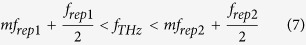


In this case, *f*_*beat1*_ is generated by photoconductive mixing between *f*_*THz*_ and (*m* + *1)f*_*rep1*_, whereas *f*_*beat2*_ is generated by photoconductive mixing between *f*_*THz*_ and *mf*_*rep2*_. The dead bandwidth *∆f*_*dead*_ is given by


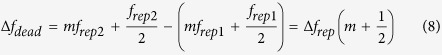


For example, when *f*_*THz*_ = 100 GHz, *f*_*rep1*_ ≈ *f*_*rep2*_ ≈ 32 MHz, ∆*f*_*rep*_* = *1.63 kHz, and *m* = 3,125, ∆*f*_*dead*_ is estimated to be around 5.09 MHz, which corresponds to 16% of the measurement window with the frequency range of *f*_*rep1*_ or *f*_*rep2*_. Figure 10(b) shows the real-time monitoring result of *f*_*THz*_ when *f*_*THz*_ was linearly tuned from 100,024,770,463 Hz to 100,044,423,685 Hz at a sweep rate of 19.653 MHz/s. One can confirm the measurement error of *f*_*THz*_ caused by the dead band.

The simplest way to reduce the dead band is to reduce *∆f*_*rep*_. There is still some room to further reduce *∆f*_*rep*_ of the dual-λ mode-locked fibre laser down to a few hundred Hz by optimizing the fibre length and dispersion. In this case, it is expected that *∆f*_*dead*_ can be reduced to around 0.5 MHz, which corresponds to 1.6% of the measurement window. Work is in progress to develop a dual-λ mode-locked fibre laser with lower ∆*f*_*rep*_.

## Conclusions

We measured the absolute frequency of CW-THz radiation using dual PC-THz combs induced by a dual-λ mode-locked fibre laser. To the best of our knowledge, this is the first time such a laser system has been employed for frequency measurement in THz region. Although this laser was operating in the free-running mode without stabilization of *f*_*rep1*_ and *f*_*rep2*_, a relative precision and accuracy of 1.2 × 10^−9^ and 1.4 × 10^−9^ were achieved at a measurement rate of 100 Hz due to the common-mode behaviour of *f*_*rep1*_ and *f*_*rep2*_, in addition to the fact that the interval between the PC-THz comb modes was kept equal regardless of the fluctuation in *f*_*rep1*_and *f*_*rep2*_. Furthermore, an abrupt or slight change in *f*_*THz*_ could be accurately monitored due to the real-time capability thanks to the use of dual PC-THz combs. Although the dual-λ mode-locked fibre laser was used in this work for measuring the frequency of CW-THz radiation in real time, it should be possible to apply it to THz spectroscopy and other metrology applications based on dual THz combs, such as ASOPS THz time-domain spectroscopy^21^,^22^,^23^,^24^, dual THz comb spectroscopy^25^,^26^,^27^, and ASOPS THz impulse ranging^28^. In particular, the constant *∆f*_*rep*_ in the free-running operation will enable correct scale conversion of the time axis or frequency axis in these spectroscopic applications. This dual-λ mode-locked fibre laser will open the door to enhance versatility and practicability in dual-THz-comb-based THz measurement systems.

## Methods

### Free-running, dual-*λ* mode-locked Er:fibre laser oscillator

As shown in Fig. 2(a), the free-running, dual-λ mode-locked Er:fibre laser oscillator consists of a 980-nm pumped laser diode (LD), a 980/1550 nm wavelength-division multiplexer (WDM), a single-mode fibre (SMF), a 2 meter length of erbium-doped fibre (EDF, Changfei 1022), a polarization-independent optical isolator (ISO), a home-made single-wall carbon nanotube saturable absorber (SWNT-SA), a fibre-squeezer-based polarization controller (PC), a 90/10 fibre output coupler (OC), and an in-line polarizer (ILP) with two 0.25-meter-long polarization maintaining fibre (PMF) pigtails at both ends. The lengths of the commercial single-mode fibres (SMF-28 and HI 1060) in the cavity were estimated to be ~3.4 m and ~0.35 m, respectively, and therefore, the total dispersion was estimated to be ~0.063 ps/nm. The SWNT-SA had a transmittance of 24% at 1540 nm and was fabricated on an FC/APC ferrule from a ~0.27 wt% SWNT solution by using the optical deposition method. By introducing the ILP with its transmission aligned along the slow axis of the PMF into the ring fibre laser, birefringence-induced filtering and loss control effects enabled multi-wavelength lasing in the cavity^12^,^13^,^19^. With the adjustment of the intracavity polarization state, simultaneous mode-locking centred on the 1530-nm and 1560-nm regions could be realized. *∆f*_*rep*_ was related to the cavity dispersion of the fibre laser, whereas *f*_*rep1*_ and *f*_*rep2*_ were related to be the fibre length; their values can be further adjusted by optimizing the fibre length and dispersion.

### Real-time determination of *f*_*THz*_ with dual PC-THz combs

Figure 5 shows a schematic diagram of the setup for measuring the frequency of CW-THz radiation The amplified *λ*_*1*_ pulse light at *f*_*rep1*_ from one EDFA (EDFA1) was collimated in free space and then focused onto a gap in a free-space-coupled, bowtie-shaped, low-temperature-grown (LT) InGaAs/InAlAs PCA (PCA1, TERA15-BT3, Menlo Systems) by a lens (L), whereas the amplified *λ*_*2*_ pulse light at *f*_*rep2*_ from the other EDFA (EDFA2) was directly fed into a fibre-coupled, dipole-shaped LT-InGaAs/InAlAs PCA detector (PCA2, TERA 15-RX-FC, Menlo Systems) via an optical fibre. This resulted in the generation of dual PC THz combs: PC-THz comb 1 with a frequency spacing *f*_*rep1*_ in PCA1 and PC-THz comb 2 with a frequency spacing *f*_*rep2*_ in PCA2.

The CW-THz test source was an active frequency multiplier chain (Millitech AMC-10-R0000 with multiplication factor = 6, tuning range = 75–110 GHz, and mean power = 2.5 mW), which amplified the output frequency of a microwave frequency synthesizer (Agilent E8257D, linewidth < 0.1 Hz) by a factor of six. Since this test source was phase-locked to a 10 MHz rubidium (Rb) frequency standard (Stanford Research Systems FS725, accuracy = 5 × 10^−11^, stability = 2 × 10^–11^ at 1 s), its output was CW-THz radiation with a linewidth of less than 0.6 Hz and a frequency accuracy similar to that of the frequency standard. When the CW-THz radiation was incident on both PCA1 and PCA2, photoconductive mixing between the CW-THz radiation and the dual PC-THz combs resulted in the output of a current signal from them. The current signals from PCA1 and PCA2 were amplified and filtered by current preamplifiers (AMP, bandwidth = 10 MHz, transimpedance gain = 10^5^ V/A), and the beat frequencies below half of *f*_*rep1*_ or *f*_*rep2*_ were extracted as *f*_*beat1*_ and *f*_*beat2*_.

Portions of light from the EDFAs were detected with photodetectors (PD, Thorlabs DET01CFC, freq. bandwidth = 1.2 GHz). Since the output signal from the PDs included a fundamental component and a series of harmonic components of *f*_*rep1*_ or *f*_*rep2*_ within the frequency bandwidth of the PDs, we selected the 30-th harmonic component of *f*_*rep1*_ or *f*_*rep2*_, namely 30*f*_*rep1*_ and 30*f*_*rep2*_, in order to magnify the frequency fluctuation. The components 30*f*_*rep1*_ and 30*f*_*rep2*_ were electrically mixed with an output signal from a local oscillator (LO, *f*_*LO*_ = 961,000,000.00 Hz) using a double-balanced mixer (M), and the resulting beat signals 30*f*_*rep1*_ − *f*_*LO*_ and 30*f*_*rep2*_ − *f*_*LO*_ were extracted by two low-pass filters (LPF). Temporal waveforms for *f*_*beat1*_, *f*_*beat2*_, 30*f*_*rep1*_ − *f*_*LO*_, and 30*f*_*rep2*_ − *f*_*LO*_ were simultaneously acquired by a digitizer (resolution = 14 bit, sampling rate = 20 MHz). From the temporal waveforms, we determined instantaneous values of *f*_*rep1*_, *f*_*rep2*_, *f*_*beat1*_, and *f*_*beat2*_ using the instantaneous-frequency-calculation algorithm involving a Fourier transform, digital frequency filtering, an inverse Fourier transform, a Hilbert transform, the time differential of the instantaneous phase, and signal averaging^8^. Finally, we determined *f*_*THz*_ by substituting these values into Eqs. (4 to 6). Since the CW-THz test source, the local oscillator, and the clock signals of the digitizer shared a common time-base signal from the frequency standard, one can evaluate the relative precision of frequency measurement without the influence of the absolute precision of the frequency standard.

## Additional Information

**How to cite this article**: Hu, G. *et al*. Measurement of absolute frequency of continuous-wave terahertz radiation in real time using a free-running, dual-wavelength mode-locked, erbium-doped fibre laser. *Sci. Rep.* **7**, 42082; doi: 10.1038/srep42082 (2017).

**Publisher's note:** Springer Nature remains neutral with regard to jurisdictional claims in published maps and institutional affiliations.
